# Enclosing a pen to improve response rate to postal questionnaire: an embedded randomised controlled trial

**DOI:** 10.12688/f1000research.23651.1

**Published:** 2020-06-09

**Authors:** Rachel Cunningham-Burley, Jenny Roche, Caroline Fairhurst, Sarah Cockayne, Catherine Hewitt, Heather Iles-Smith, David J. Torgerson, SSHeW Study Team

**Affiliations:** 1 York Trials Unit, Department of Health Sciences, University of York, York, YO10 5DD, UK; 2 Research and Innovation Department, The Leeds Teaching Hospitals NHS Trust, Leeds, LS9 7TF, UK

**Keywords:** randomised controlled trial, embedded trial, postal questionnaire, response rate, pen

## Abstract

**Background:** Poor response to questionnaires collecting outcome data in randomised controlled trials (RCTs) can affect the validity of trial results. The aim of this study within a trial (SWAT) was to evaluate the effectiveness of including a pen with a follow-up postal questionnaire on response rate.

**Methods:** A two-armed RCT was embedded within SSHeW (Stopping Slips among Healthcare Workers), a trial of slip-resistant footwear to reduce slips in NHS staff.  Participants were randomised 1:1 to receive a pen or no pen with their follow-up questionnaire. The primary outcome was the proportion of participants who returned the questionnaire. Secondary outcomes were: time to response, completeness of response, and whether a postal reminder notice was required. Data were analysed using logistic regression, linear regression and Cox proportional hazards regression.

**Results:** Overall, 1466 SSHEW trial participants were randomised into the SWAT. In total, 13 withdrew from the host trial before they were due to be sent their follow-up questionnaire, 728 participants received a pen with their questionnaire, and 725 did not receive a pen.  A questionnaire was returned from 67.7% of the pen group and 64.7% of the group who did not receive a pen. There was no significant difference in return rates between the two groups (OR 1.15, 95% CI 0.92 to 1.43, p=0.22), nor level of completeness of the questionnaires (AMD -0.01, 95% CI 0.06 to 0.05, p=0.77).  There was weak evidence of a reduction in the proportion of participants requiring a reminder and in time to response in the pen group.

**Conclusion: **Inclusion of a pen with the follow-up postal questionnaire sent to participants in the SSHeW trial did not statistically significantly increase the response rate. These results add to the body of evidence around improving response rates in trials.

**Trial registration:** ISRCTN 33051393 (for SSHEW). Registered on 14/03/2017.

## Introduction

Randomised controlled trials (RCTs) are key to evaluating the effectiveness of interventions and often use postal questionnaires to collect outcome data. However, low response rates can limit the validity of the trial findings by reducing the power of the study and introducing bias ^1^.

Numerous strategies to increase response rates have been studied ^2, 3^ including sending a pen with the questionnaire. The pen acts both as a facilitator to aid completion of the questionnaire, and an incentive to return it ^4, 5^. The effectiveness of this intervention is equivocal with some studies reporting an increase in response rate ^5– 7^ whilst others failed to show a positive impact ^4, 8^. These studies displayed considerable heterogeneity and only two were embedded in RCTs ^6, 7^. A Study within a Trial (SWAT) is a self-contained study embedded within a host trial that can be used to evaluate strategies designed to improve trial efficiency ^9^. This SWAT evaluated the effectiveness of enclosing a pen with a follow-up postal questionnaire on response rates in the SSHeW trial ^10^.

## Methods

### Design

This two-armed RCT was embedded in the SSHeW trial, a trial evaluating the effectiveness of slip-resistant footwear to reduce slips in NHS staff ^10^. The SSHeW trial was registered (ISRCTN 33051393) and the trial protocol has been published ^10^.

### Participants

The SWAT was conducted in seven NHS Trusts in England and included all eligible participants in the SSHeW trial who were due to be sent their 14-week postal questionnaire between 04.07.2018 and 12.02.2019.

### Intervention

The intervention group were sent a York Trials Unit, University of York branded pen with their questionnaire. The control group did not receive a pen.

### Outcomes

The SWAT outcomes are outlined in Table 1.

**Table 1.** SWAT outcomes.

Outcome	Type	Definition
Proportion of participants who return questionnaire (Primary Outcome)	Binary (returned/not returned)	Proportion of 14-week questionnaires returned to York Trials Unit. (Returns were censored at 11.06.2019)
Time to response	Time to event (days)	Number of days between the date the 14-week questionnaire was sent and the date the returned questionnaire was received by York Trials Unit.
Completeness of response	Continuous (0–5)	Number of completed responses to 5 key questions on the 14-week questionnaire.
Reminder notice sent	Binary (sent/not sent)	Proportion of participants sent a reminder questionnaire (sent three weeks after the initial questionnaire if no response had been received, no additional pens were sent with reminders).
Cost	Continuous	Consideration of cost effectiveness of pen inclusion

### Sample size

As is usual with an embedded trial, a formal sample size calculation was not undertaken as the sample size was determined by the number of participants due to receive their 14-week questionnaire.

We anticipated that randomising 2,000 participants into the SWAT would provide 80% power to detect an absolute difference of 6% (two-sided α=0.05) in response rates between the two groups, assuming a control rate of 60%.

### Randomisation

Participants were allocated to either the intervention (pen) or control (no pen) group using simple randomisation in a 1:1 ratio. The allocation sequence was generated by the SSHeW trial statistician, who was not involved in sending out the questionnaires.

### Blinding

Participants were not aware of their involvement in this SWAT but due to the nature of the intervention participants and study team members could not be blinded to group allocation.

### Approvals

This SWAT was approved by the Department of Health Sciences Research Ethics Committee at the University of York and the Health Research Authority (HSRGC/2016/187/A).

### Statistical analysis

Data were analysed using Stata version 15 ^11^ on an intention-to-treat basis, using two-sided tests at the 5% significance level. The models used for each outcome are given in Table 2, the values associated with the pen allocation from each model is presented with its 95% confidence interval and p-value. All models were adjusted for main trial group allocation (slip-resistant footwear or wait-list control) and pen sub-study allocation (pen or no pen).

**Table 2.** Analysis models.

Outcome	Analysis model	Value presented
Proportion of participants who return questionnaire	Logistic regression	Odds ratio (OR)
Time to response	Cox proportional hazards regression	Hazard ratio
Completeness of response	Linear regression	Adjusted mean difference
Reminder notice sent	Logistic regression	OR

### Costing

The total cost of a standard SSHeW questionnaire pack was £2.42 (envelope and postage: £0.86; questionnaire and cover letter: £0.65; pre-paid envelope and postage: £0.91). The additional cost of including a pen was £0.32. The cost analysis incorporates the changes in number of questionnaires returned and reminders required.

## Results

**Figure 1.**
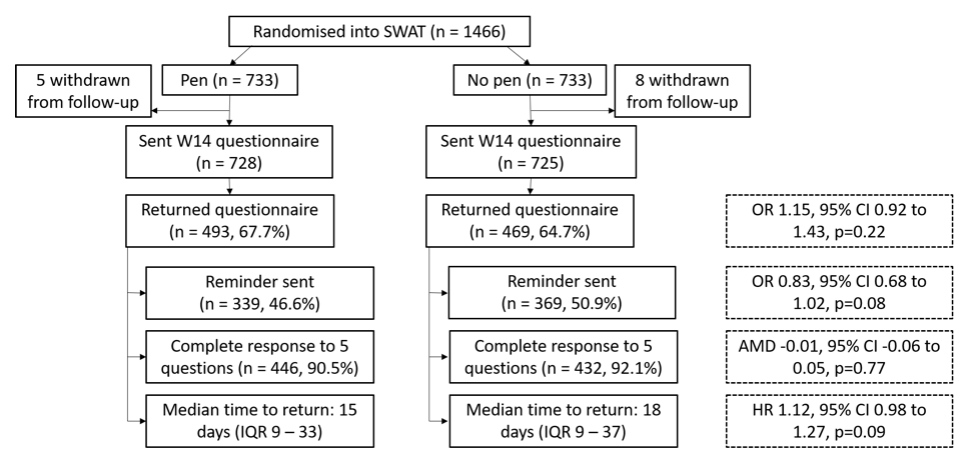
CONSORT flow diagram of participants in the embedded trial.

A total of 1466 participants were included in the SWAT (pen, n=733; no pen, n=733). In total, 13 participants withdrew from the main SSHeW trial after they had been randomised into the SWAT but before being sent their follow-up questionnaire, leaving 1453 participants ( Figure 1). Baseline characteristics are summarised descriptively in Table 3.

**Table 3.** Baseline characteristics of participants included in the analysis.

	Pen (n = 728)	No pen (n = 725)	Overall (n = 1453)
**Main trial allocation, n (%)** Usual Care Intervention	355 (48.8) 373 (51.2)	376 (51.9) 349 (48.1)	731 (50.3) 722 (49.7)
**Age (years),** **mean (SD)**	43.0 (11.1)	42.9 (11.5)	43.0 (11.3)
**Gender, n (%)** Male Female Prefer not to say	111 (15.3) 616 (84.6) 1 (0.1)	90 (12.4) 635 (87.6) 0 (0.0)	201 (13.8) 1251 (86.1) 1 (0.1)
**Job role, n (%)** Admin and IT Facilities Direct patient care Other	44 (6.0) 50 (6.9) 610 (83.8) 24 (3.3)	51 (7.0) 38 (5.2) 614 (84.7) 22 (3.0)	95 (6.5) 88 (6.1) 1224 (84.2) 46 (3.2)
**Average working hours, mean (SD)**	35.0 (5.2)	35.1 (4.9)	35.0 (5.0)
**Injury resulting from a slip or fall** **(in previous 12 months), n (%)**	43 (5.9)	30 (4.1)	73 (5.0)

Results are presented in Table 4. Overall, 962 (66.2%) questionnaires were returned (pen, 67.7%; no pen, 64.7%) and an average of 4.9/5 items were completed. There was no evidence of a difference in return rate between the groups (OR 1.15, 95% CI 0.92 to 1.43, p=0.22), nor number of items completed (AMD -0.01, 95% CI 0.06 to 0.05, p=0.77).

**Table 4.** Summary of results. OD, odds ratio; HR, hazards ratio; AMD, adjusted mean difference

Results					
Returns, n/total (%)			OR	95% CI	p-value
Pen	No pen	Overall			
493/728 (67.7)	469/725 (64.7)	962/1453 (66.2)	1.15	0.92, 1.43	0.22
Time to response (days), median (IQR)			HR	95% CI	p-value
Pen	No pen	Overall			
15 (9-33)	18 (9-37)	16 (9-35)	1.12	0.98, 1.27	0.09
Completeness of response, mean (SD)			AMD	95% CI	p-value
Pen	No pen	Overall			
4.9 (0.4)	4.9 (0.4)	4.9 (0.4)	-0.01	-0.06, 0.05	0.77
Reminder sent, n/total (%)			OR	95% CI	p-value
Pen	No pen	Overall			
339/728 (46.6)	369/725 (50.9)	708/1453 (48.7)	0.83	0.68, 1.02	0.08

**Figure 2.**
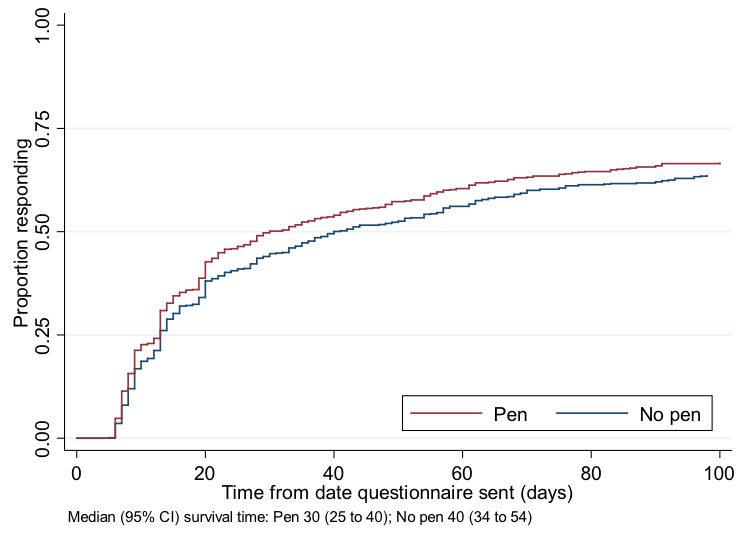
Kaplan-Meier curve for time to questionnaire return.

There was weak evidence of a difference, in favour of the pen group, in both time to return (median time to return 15 vs 18 days; HR 1.12, 95% CI 0.98 to 1.27, p=0.09) ( Figure 2), and in the proportion of participants requiring a reminder (OR 0.83, 95% CI 0.68 to 1.02, p=0.08).

### Costing

A 3% difference in questionnaire response rate and an absolute difference in the percentage of participants who required a reminder of 1.1% were found. Considering these to be true effects, in order to receive one additional questionnaire, 33 participants would have to be sent a pen, at a cost of approximately 33x32p=£10.56. Approximately 91 participants would need to be sent a pen to prevent one reminder mailing and therefore to save £2.42. Hence, roughly one reminder is required per three retained participants, and the cost per retained participant is approximately £10.

## Discussion

Whilst the results of all outcomes in this SWAT favoured the pen group, we found that the addition of a pen did not statistically significantly increase the response rate to, or completeness of, a follow-up questionnaire sent at 14 weeks post-randomisation among participants of the SSHeW trial. There was some evidence of a reduction in time to response and the number of reminders required.

It may be that, in this group of participants, the pen failed to act as a facilitator or was not a sufficient incentive to return the questionnaire, given the fact that participants in the trial already received a free pair of shoes (although offer of shoes was not conditional on returning the questionnaire).

However, the trial ultimately only had about 40% power to detect a difference of 3% in response rates (from 64.7 to 67.7%) and is therefore at risk of a type II error. Another potential weakness is that, due to the select population of healthcare workers, the results may not be generalisable to other populations or contexts.

The strength of this study is that it was a randomised trial.

## Conclusion

This SWAT suggests that enclosing a pen in a questionnaire mail out may be an effective method to increase response rates but was likely underpowered to detect a statistically significant difference of the 3% observed. Since pens are inexpensive, even a small difference is likely to be cost-effective. The results contribute to the body of evidence regarding this intervention and may be included in future meta-analyses to improve power.

## Data availability

### Underlying data

Open Science Framework: SSHeW Trial Pen SWAT, https://doi.org/10.17605/OSF.IO/YQ76U ^12^.

### Reporting guidelines

Open Science Framework: CONSORT checklist for ‘Enclosing a pen to improve response rate to postal questionnaire: an embedded randomised controlled trial’, https://doi.org/10.17605/OSF.IO/YQ76U ^12^.

Data are available under the terms of the Creative Commons Zero "No rights reserved" data waiver (CC0 1.0 Public domain dedication).
