# Supplementary material for: Enclosing a pen to improve response rate to postal questionnaire: an embedded randomised controlled trial
Source: F1000Res. 2020 Jun 9;9:577. [Version 1] doi: 10.12688/f1000research.23651.1 (PMC7941094; doi:10.12688/f1000research.23651.1)
Supplement: Peer review history

# Peer review: Reviewer response for version 1

**DOI:** 10.5256/f1000research.26098.r80788

**Published:** 2021-03-08

**Authors:** Karen Bracken

**Affiliations:** 1 NHMRC Clinical Trials Centre, The University of Sydney, Sydney, NSW, Australia

This paper describes the result of a SWAT embedded within the SSHeW trial. The SWAT aimed to evaluate whether including a pen with a follow-up postal questionnaire would impact questionnaire response rate. The authors indicate that previous studies have yielded mixed results. The results of this SWAT add to the body of existing evidence.

 The paper is clear and concise and addresses a common problem faced by trials; low response rates to questionnaires. The paper provides adequate detail for other trialists to replicate the intervention within their own trial. The inclusion of cost analyses is particularly useful.

 I was interested to see that the cost of including the pen was very low and would have liked an explanation of the components of the cost (cost of pen, additional cost of postage and posting materials).

 Like many SWATs, this SWAT was unavoidably constrained in sample size by the host trial, making conclusions difficult due to lack of power. It is unclear from the paper whether the authors expected that the sample size of 1466 was sufficient to detect an operationally important difference between groups. If not, then I suggest the conclusion be updated to make this clear, rather than stating that a statistically significant increase was not found. Would the authors suggest that further SWATs are needed in this area based on the Trial Forge guidance? (https://trialsjournal.biomedcentral.com/articles/10.1186/s13063-019-3980-5 ^1^)

 Also, I suggest removing the observed power calculation from the discussion (https://lesslikely.com/statistics/observed-power-magic/ provides an explanation as to why observed power calculations should be avoided).

Is the work clearly and accurately presented and does it cite the current literature?

Yes

If applicable, is the statistical analysis and its interpretation appropriate?

Partly

Are all the source data underlying the results available to ensure full reproducibility?

Yes

Is the study design appropriate and is the work technically sound?

Yes

Are the conclusions drawn adequately supported by the results?

Partly

Are sufficient details of methods and analysis provided to allow replication by others?

Yes

Reviewer Expertise:

Trial operations and recruitment

I confirm that I have read this submission and believe that I have an appropriate level of expertise to confirm that it is of an acceptable scientific standard.

## References

[1] : Trial Forge Guidance 2: how to decide if a further Study Within A Trial (SWAT) is needed. *Trials*.2020;21(1) : 10.1186/s13063-019-3980-5 33 10.1186/s13063-019-3980-5 31910861PMC6945587

# Peer review: Reviewer response for version 1

**DOI:** 10.5256/f1000research.26098.r68921

**Published:** 2020-09-02

**Authors:** Hanne Bruhn, Anna Duncan, Magaly Aceves Martins

**Affiliations:** 1 CHaRT, University of Aberdeen, Aberdeen, UK; 2 HSRU, University of Aberdeen, Aberdeen, UK; 3 Health Services Research Unit, University of Aberdeen, Aberdeen, UK

The current study is a SWAT – a Study Within A Trial. A SWAT is a methodological study aiming to add to the evidence base for randomised controlled rial design and conduct. In a SWAT an intervention to improve the conduct of trials is tested in an ongoing ‘host’ trial.

 The current host trial was Stopping Slips among Healthcare Workers (SSHeW), a trial testing slip-resistant footwear with NHS Staff to reduce slips at work. The SWAT intervention was to include a pen with the follow-up questionnaire to test whether participants who received a pen were more likely to return their questionnaire than those participants who did not receive a pen. The authors also looked at whether inclusion of a pen with the questionnaire affected the time taken to send back the questionnaire; how complete they questionnaire was answered and; whether a reminder had to be sent for return of the questionnaire. The SWAT was run on the 14 week post randomisation follow-up questionnaire. All SSHEW participants were randomised into the SWAT (n=1466). There were no significant difference in return rates between the two groups but weak evidence that including a pen might make participants return the questionnaire sooner and less will need a reminder.

 This study reports findings contributing to the evidence base for how trial teams can increase questionnaire response rates in their trials, using the gold standard randomised comparison. This is a hugely important area as low response rates affect the conclusions that can be drawn from trial findings. Unfortunately, low questionnaire response rates are something which continues to affect a lot of trials.

 We have a few comments which should be addressed:

 ABSTRACT

 No comments.

 INTRODUCTION

 It would be interesting to know if the authors expected there to be an issue with the questionnaire return rate in the host trial and what was the basis of this expectation (e.g. low response rate to previous questionnaires)? Please consider adding what the expected response rate for the host trial was. This could be added in the introduction or methods according to the authors preference. Commonly, SWATs are reactive so some background information to the approach in this SWAT would provide a helpful context.  

 The authors mention that the pen would act as facilitator and incentive, it would be helpful to have any reference to this use as a behavioural motivator or barrier. 

 METHODS

 It would be interesting to know if the investigators found any issues with posting a pack including a pen, eg additional bulk causing any issues with standard franking machines. Did any changes have to be made to the process of posting out to include the pen?

 RESULTS

 No comments.

 DISCUSSION

 Please include some consideration as to whether the timing of the incentive to return questionnaires might have affected the findings. In many trials participants are followed-up for two years or longer and that is the point when it gets really tough to keep the response rate high (reviewers’ personal experiences). It would be really valuable to have the authors thoughts on this for trial teams looking for help.

 Paragraph 3 -The authors list a lack of power as a limitation. We’d suggest highlighting that many SWATs will be underpowered because they are generally unable to change the size of the host trial; they are made for meta-analysis really.

   

 CONCLUSION

 This is a very well conducted and clearly written article on a SWAT that investigated including a pen  to improve response rates to a questionnaire, and importantly addressed the cost effectiveness of the intervention as one of the outcomes.

 The results of the SWAT did not provide strong evidence in itself of the effectiveness of including a pen to improve response rates but the results add to the body of evidence and  will be important to include in meta-analysis to examine the effectiveness of a pen as an incentive/facilitator in retention rates.

 While the pen itself is inexpensive, when it is multiplied up over the issue of thousands of questionnaires is an additional cost any trials unit would have to justify to funder. The cost for a 3% gain is £10 per retained participant according to statement in Results (Costings) so not an insubstantial amount for weak evidence of an effect. Replication of this SWAT is needed to include other settings, different timepoints etc before concluding that including a pen may be effective and cost-effective way of improving response rates. The authors should expand on this in the concluding paragraph and consider revising sentence 2 of the concluding paragraph.

Is the work clearly and accurately presented and does it cite the current literature?

Yes

If applicable, is the statistical analysis and its interpretation appropriate?

I cannot comment. A qualified statistician is required.

Are all the source data underlying the results available to ensure full reproducibility?

Yes

Is the study design appropriate and is the work technically sound?

Yes

Are the conclusions drawn adequately supported by the results?

Partly

Are sufficient details of methods and analysis provided to allow replication by others?

Yes

Reviewer Expertise:

Hanne Bruhn: Health Services Research and Trial Methodology. Anne Duncan: Trial Management and conduct and Trial Methodology. Magaly Aceves Martins: Nutrition and systematic reviewing.

We confirm that we have read this submission and believe that we have an appropriate level of expertise to confirm that it is of an acceptable scientific standard.
